# Exploring the relationship between epigenetic DNA methylation and cardiac fibrosis through Raman microspectroscopy

**DOI:** 10.1152/ajpcell.00209.2023

**Published:** 2023-06-19

**Authors:** Lucas Becker, Ivonne A. Montes-Mojarro, Shannon Lee Layland, Ali Nsair, Falko Fend, Julia Marzi, Katja Schenke-Layland

**Affiliations:** ^1^Department for Medical Technologies and Regenerative Medicine, Institute of Biomedical Engineering, University of Tübingen, Tübingen, Germany; ^2^Cluster of Excellence iFIT (EXC 2180) “Image-Guided and Functionally Instructed Tumor Therapies”, University of Tübingen, Tübingen, Germany; ^3^Division of Cardiology, Department of Medicine, Cardiovascular Research Laboratories, David Geffen School of Medicine at UCLA, Los Angeles, California, United States; ^4^Institute of Pathology and Neuropathology, University Hospital Tübingen, Tübingen, Germany; ^5^NMI Natural and Medical Sciences Institute at the University Tübingen, Reutlingen, Germany

**Keywords:** collagen, extracellular matrix, non-destructive imaging, pathological tissue remodeling, Raman spectroscopy

## Abstract

Cardiomyopathies are associated with fibrotic remodeling of the heart, which is characterized by the excessive accumulation of collagen type I (COL I) due to chronic inflammation and suspected epigenetic influences. Despite the severity and high mortality rate of cardiac fibrosis, current treatment options are often inadequate, underscoring the importance of gaining a deeper understanding of the disease’s underlying molecular and cellular mechanisms. In this study, the extracellular matrix (ECM) and nuclei in fibrotic areas of different cardiomyopathies were molecularly characterized by Raman microspectroscopy and imaging and compared with the control myocardium. Patient samples were obtained from heart tissue affected by ischemia, hypertrophy, and dilated cardiomyopathy and analyzed for fibrosis through conventional histology and marker-independent Raman microspectroscopy (RMS). Prominent differences between control myocardium and cardiomyopathies were revealed by spectral deconvolution of COL I Raman spectra. Statistically significant differences were identified in the amide I region of spectral subpeak at 1,608 cm^−1^, which is a representative endogenous marker for alterations in the structural conformation of COL I fibers. Moreover, epigenetic 5mC DNA modification was identified within cell nuclei by multivariate analysis. A statistically significant increase in signal intensities of spectral features indicative of DNA methylation was detected in cardiomyopathies in accordance with immunofluorescence 5mC staining. Overall, RMS is a versatile technology in the discrimination of cardiomyopathies based on molecular evaluation of COL I and nuclei while providing insights into the pathogenesis of the diseases.

**NEW & NOTEWORTHY** Cardiomyopathies are associated with severe fibrotic remodeling of the heart, which is characterized by the excessive accumulation of collagen type I (COL I). In this study, we used marker-independent Raman microspectroscopy (RMS) to gain a deeper understanding of the disease’s underlying molecular and cellular mechanisms.

## Graphical abstract

**Figure:**
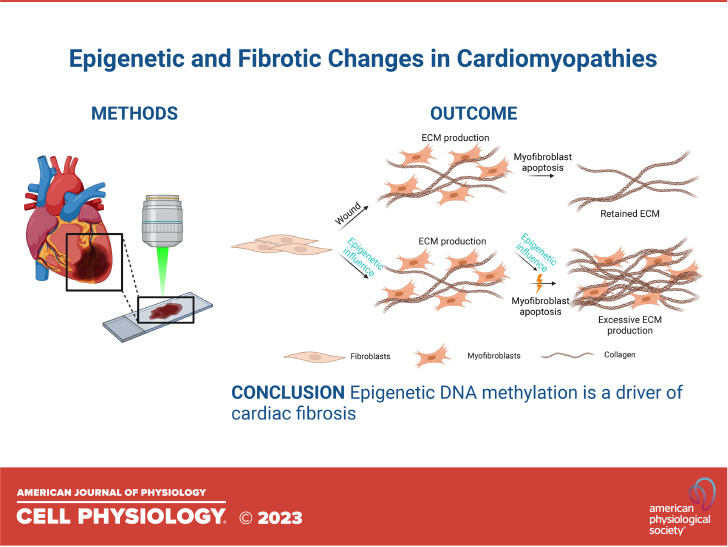

## INTRODUCTION

Cardiomyopathies comprise a heterogeneous group of myocardial diseases characterized by mechanical or electrical dysfunction (1). Myocardial infarction is defined as a muscle failure caused by a blocked blood vessel (ischemia) resulting in an anoxic state of the heart muscle (2). Spasms of the coronary artery, restricting the arterial lumen (3) or intravascular aggregation of lipids, and white blood cells inducing myocardial damage by inflammatory processes (4) are additional causes of ischemia. Most common primary diseases are diabetes, hypertension, hyperlipidemia, overweight, and obesity (5, 6). Although genetic predisposition plays a minor role in pathological development, epigenetic alterations were linked to fibrotic tissue modification in ischemia (7). Epigenetics refer to the mechanisms that result in heritable changes in the structure and function of chromatin without modifying the underlying DNA nucleotide sequences (8). Epigenetic mechanisms include, for example, transcriptional silencing of methylated DNA regions by DNA methylation, opening or closing of chromatin by histone modifications, initiation of repair mechanisms, or elongation of transcription (9). The initiation of these mechanisms is mainly based on reversible changes in DNA in the form of methylation of cytosine and histone changes such as methylation, acetylation, phosphorylation, or ubiquitination (10).

In contrast, cardiac hypertrophy is an adaptive process of the myocardium in response to a variety of intrinsic and extrinsic stimuli, including left ventricular dilation, decreased systolic function, cardiomyocyte loss, and the development of fibrosis ultimately leading to heart failure and death (11, 12). Finally, dilatated cardiomyopathy is characterized by the presence of left ventricular dilatation and contractile dysfunction. Common causes of dilated cardiomyopathy are genetic mutations, or myocarditis (13).

The determining feature of these cardiomyopathies is the appearance of fibrotic tissue. In normal wound healing, fibroblasts are activated and transform into myofibroblasts. Hallmarks of myofibroblasts are the expression of contractile α smooth muscle actin (αSMA) and the secretion of extracellular matrix (ECM) proteins such as collagen type I (COL I) (14). Normally, myofibroblasts are removed by apoptotic pathways after completion of the wound healing process, however, epigenetic modifications could lead to resistance mechanisms, resulting in an excessive production of COL I (14).

Clinical evaluation of fibrosis currently relies primarily on the examination of tissue biopsies by standard histological analyses (H&E and Masson trichrome staining) or imaging techniques such as computed tomography, magnetic resonance imaging, and ultrasound elastography (15–18). In contrast, epigenetic evaluation often requires the disruption of tissue and the isolation of cellular DNA to perform next-generation sequencing (NGS) or methylation microarrays (19, 20). In recent years, noninvasive spectroscopic methods such as Raman microspectroscopy (RMS) have emerged as an alternative to conventional staining protocols (21–24). Recently, our group used Raman microspectroscopy to identify epigenetic 5mC alterations in human colon carcinoma cells (25). Furthermore, it has recently been demonstrated that fibrotic collagen changes can be identified by spectral deconvolution in various human tissues (22).

In this study, RMS was used to monitor collagen-rich regions of ex vivo tissues of cardiomyopathies including ischemic heart disease (IHD), hypertropic cardiomyoopathy (HCM), and dilatative cardiomyopathy (DCM). COL I spectra were extracted and underwent spectral deconvolution as well as peak-filter and image-based ratio analysis, whereas nuclei spectra were analyzed by principal component analysis (PCA) in comparison with 5mC immunofluorescence (IF) imaging.

## MATERIALS AND METHODS

### Collection of Human Tissue Samples

Formalin-fixed paraffin-embedded (FFPE) blocks were collected from patients undergoing medically indicated orthotopic heart transplantation at the University Department of General, Visceral and Transplant Surgery at the University of California (UCLA). Cases with different cardiomyopathies where fibrosis is accumulated in tissues were investigated including cases diagnosed as IHC, HCM, and DCM. For comparison, human control tissue from myocardium without fibrosis was analyzed (*n* = 5). All samples were collected after informed consent. The study was approved by the local ethical committee at UCLA. A microtome (MICROM HM560, Thermo Scientific, Waltham, MA) was used to obtain serial 10-µm cross sections of the tissue. A pathologist evaluated all tissues and confirmed the location of fibrotic areas using routine Masson trichrome staining.

### Masson Trichrome Staining

Tissue sections were stained by utilizing the automatic slide stainer Tissue Tek Prisma (Sakura, Finetek) following the manufacturer’s protocol. The sections were scanned and imaged using an Axio Observer (Carl Zeiss Microscopy GmbH, Oberkochen, Germany) at ×63 magnification.

### Deparaffinization

FFPE tissue sections were deparaffinized using a modified protocol where samples were subjected to a thermal treatment at 60°C for 10 min, followed by three sequential incubations in xylol for 10 min each. The samples were then incubated in a series of ethanol solutions with progressively lower concentrations.

### Picrosirius Red Staining

Weigert’s hematoxylin was applied for 8 min and washed with tap water for 10 min to stain nuclei. Afterward, tissue sections were treated with 0.1% picrosirius red solution (Morphisto, Frankfurt/Main, Germany) for 60 min and washed with 0.5% acetic acid and 100% ethanol for 5 min. Picrosirius red (PSR)-stained heart tissues were imaged by polarized light microscopy utilizing an Axio Observer at ×63 magnification. Alterations in the amounts of red/orange and yellow/green were recorded. To identify red, orange, yellow, and green fibers, the images were transferred to Hue, Saturation, Value (HSV) color space in MATLAB 2019 b. The percentages of red (0–9, 230–256), orange (10–38), yellow (39–51), and green (52–128) pixels were analyzed for each image. Three images were analyzed for each sample. PSR images were additionally used to identify collagen fiber alignment. Angles of collagen were measured with ImageJ (Fiji version 2.0.0, National Institutes of Health, NIH). Utilizing the “OrientationJ” plugin collagen fiber coherency was defined for each image. In brief, coherency is calculated as the ratio between the difference and the sum of tensor eigenvalues describing the directionality of fibers (26).

### COL I and αSMA Immunofluorescence Staining

Antigen retrieval was performed with Tris-EDTA buffer (pH 9, 0.05%) and citrate buffer (pH 6) followed by treatment of goat serum block solution (2%). Tissue sections were then incubated overnight with the following primary antibodies: rabbit polyclonal anti-Collagen I (6.6 mg/L; Acris, Herford, Germany) and mouse IgG2a monoclonal anti-α smooth muscle actin (αSMA) (2 mg/L; Sigma-Aldrich). Secondary antibodies AlexaFluor 488 conjugated goat anti-rabbit (4 mg/L; Thermo Fisher Scientific Life Technologies) and AlexaFluor 594 conjugated goat anti-mouse (4 mg/L, Thermo Fisher Scientific Life Technologies, Sindelfingen, Germany) were used as fluorescence labels. Afterward, Drag5 (5 μM; BioLegend, San Diego) was applied to the tissue sections for 15 min to stain nuclei. IF stains were imaged utilizing a Zeiss LSM 880 (Zeiss microscopy GmbH). Images were acquired through a C Plan-Apochromat 63x/1.4. Oil DIC UV-VIS-IR M27 objective at a bit depth of 16 bit, scaling of 0.07 × 0.07 µm per pixel, and image size of 476 × 476 pixel, resulting in 33.74 × 33.74 µm large images. Image analysis was performed using ImageJ. For each tissue, three images were analyzed.

### 5mC Immunofluorescence Staining

Antigen retrieval was performed with Tris-EDTA buffer (pH 9, 0.05%) and citrate buffer (pH 6) followed by treatment of goat serum block solution (2%). Tissue sections were then placed in 88% of methanol for 30 min. After washing twice with DPBS for 10 min, 1 M HCl was applied for 90 min. Slides were then neutralized with 0.1 M sodium tetraborate (pH = 8.5) for 30 min. Then, unspecific binding sites were blocked with a 2% goat-blocking solution for 30 min. Tissue sections were then incubated overnight with the following primary antibodies: 5mC mouse monoclonal IgG primary antibody (1 µg/mL, Sigma Aldrich) and rabbit polyclonal anti-Collagen I (6.6 mg/L) at 4°C overnight. Secondary antibodies AlexaFluor 488 conjugated goat anti-rabbit (4 mg/L) and AlexaFluor 594 conjugated goat anti-mouse (4 mg/L) were used as fluorescence labels. After washing twice with DPBS for 10 min, autofluorescence was quenched with Vector TrueView autofluorescence quenching Kit (Vektor Laboratories, Newark). Afterward, Drag5 (5 μM) was applied on the tissue sections for 15 min to stain nuclei before mounting with ProLong gold antifade reagent. IF images were acquired utilizing a Zeiss LSM 880 at ×63 magnification. Analysis was performed using ImageJ. For each tissue, three images were analyzed.

### Raman Microspectroscopy

RMS measurements of tissue sections were performed with a customized confocal Raman microspectrometer (WITec alpha 300 R, Ulm, Germany) equipped with a 532 nm laser, a charge-coupled device (CCD) camera, and a 600 g/mm grating. Prior to Raman measurement, heart tissue samples were deparaffinized and kept humid with PBS during the entire measurement. Three Raman maps per sample with a size of 100 × 100 µm at a resolution of 1 µm/pixel at an integration time of 0.05 s were acquired utilizing a ×63 apochromat water dipping objective (N.A. 1.0; Carl Zeiss Microscopy GmbH). For all measurements, the laser power was set to 50 mW.

### Spectral Analysis

All Raman maps were subjected to cosmic ray removal, polynomial baseline correction, cropping to 400–3,000 cm^−1^, and area intensity normalization with the software WITec project 5 (WITec GmbH). True component analysis (TCA) was used to decompose Raman maps into five major spectral components including nuclei, COL I, COL III, myosin, and αSMA to generate false color-coded Raman images.

### PCA

For in-depth analysis of differences in control myocardium and different cardiomyopathies, PCA was performed on 200 extracted spectra from nuclei and COL I per patient with Unscrambler software (Unscrambler X10.5, CAMO, Oslo, Norway).

### Spectral Deconvolution

Spectral deconvolution was performed as previously described (22). In short, with the software WITec project 5, spectral deconvolution was used to calculate the peak width and peak area of the substructural bands of the amide I region (1,550–1,720 cm^−1^) of COL I. Collagen maps were extracted from the Raman data and then cropped to the amide I region before normalization to the peak at 1,667 cm^−1^ to 1. Inside the fitting region between 1,508 and 1,780 cm^−1^ the initial position of calculations was set to five wavenumbers based on the shape of the amide I region located at 1,563, 1,588, 1,608, 1,637, and 1,667 cm^−1^. For spectral deconvolution, the Lorentz fitting algorithm was chosen, with a maximum number of iterations of 1,000, whereas fitting five functions (27, 28). Furthermore, peak intensity ratios of averaged COL I spectra were calculated by division of the maximum of the amide I peak located at 1,667 cm^−1^ by the intensity at 1,608 cm^−1^.

### Raman Image Ratio Analysis

To provide information on peak ratios of all individual collagen spectra within one Raman map, sum filter images of collagen maps were created using WITec project 5 software as previously described (22). Sum filter images were collected at 1,667 ± 25 cm^−1^ and at 1,608 ± 10 cm^−1^ and then divided before exporting them to MATLAB 2019 b. The obtained filter images were subjected to histogram analysis showing the distribution of all individual ratios. The ratios were rounded to integers before mode analysis.

### Statistical Analysis

Statistical comparisons were performed from a minimum of five independent patient samples per pathology. Statistical analysis was performed using GraphPad Prism version 9.00 (GraphPad Software). Results are shown throughout the entire manuscript as mean ± standard deviation. All *n* numbers, applied tests, and corresponding significance for each result are listed in the figure legends.

## RESULTS

### Histological Staining Identifies Fibrotic Lesions

Fibrosis is a condition characterized by excessive collagen formation, leading to tissue remodeling and the development of persistent scar tissue, although the reason for this overproduction may vary in different cardiomyopathies (29, 30). Therefore, histological stains such as Masson’s trichrome staining are utilized to diagnose and localize fibrotic lesions by visualizing excessive amounts of collagen fibers. In this study, the control tissue of myocardium was compared with fibrotic pathologies of myocardium derived from coronary ischemia, hypertrophy, and dilatation to identify structural and molecular patterns describing fibrotic collagen fibers.

**Figure 1.**
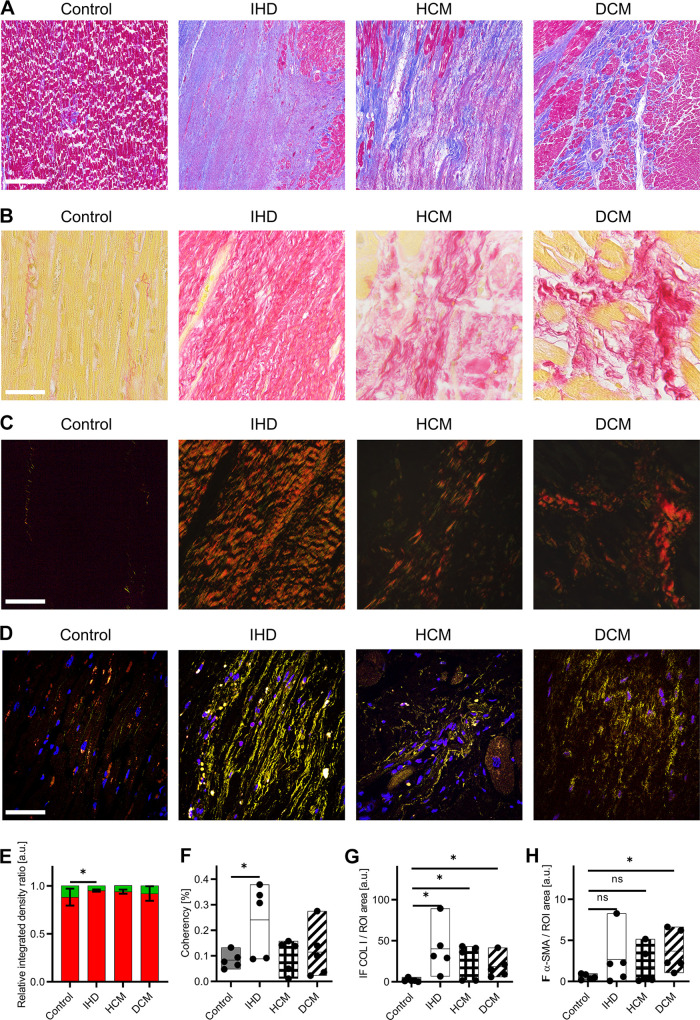
Histological staining allows the localization of fibrosis. *A*: Masson’s trichrome staining of control myocardium, ischemic heart disease (IHD), hypertrophic cardiomyopathy (HCM), and dilated cardiomyopathy (DCM) identify fibrosis by excessive collagen staining in blue. Scale bar = 500 µm. *B*: Picrosirius red (PSR) staining under brightfield illumination of control myocardium, IHD, HCM, and DCM. Scale bar = 100 µm. *C*: PSR staining under polarized light of control myocardium, IHD, HCM, and DCM. Scale bars = 100 µm. *D*: immunofluorescence (IF) images of control myocardium, IHD, HCM, and DCM show the complexity of the tissues. Colors in IF staining: Nuclei (blue), COL I (yellow), and αSMA (red). Scale bar = 100 µm. *E*: quantification of red collagen and green collagen fibers. *F*: coherency analysis shows parallel aligned fibers only in IHD. *G*: quantification of the amount of COL I based on IF images normalized by the whole tissue area. *H*: quantification of the amount of αSMA based on IF images normalized by the whole tissue area. Statistical analysis: *t* test, *n* = 5, *P < 0.05, ns: not significant.

Figure 1*A* shows exemplary Masson’s trichrome stains of control myocardium, IHD, HCM, and DCM. Although control myocardium displayed scattered network-forming collagen fibers depicted in blue between muscles, infarcted fibrotic lesions are identified by excessive amounts of interstitial collagens in IHD, HCM, and DCM.

### Collagen Fiber Maturity and Orientation Differs across Cardiomopathies

Picrosirius red (PSR) staining of tissues allowed the assessment of collagen maturity due to their birefringent characteristics. In Fig. 1*B*, exemplary PSR images under bright-field illumination of control myocardium, and the infarcted conditions of IHD, HCM, and DCM are displayed with collagen fibers stained in red. Corresponding polarized light images of the respective areas are displayed in Fig. 1*C*. Color threshold analysis of the percentage of red/orange collagen fibers and yellow/green collagen fibers varied strongly between the different heart diseases (Fig. 1*E*). Although the ratio of control and DCM showed no statistically significant difference (*P* = 0.3202), IHD exhibited significantly more red/orange fibers compared with the control (*P* = 0.0403). In HCM, the tendency of increased amount in red/orange fibers was detected (*P* = 0.0851). Conclusive differences between control and fibrotic heart tissues were not identified between the investigated samples. The quantification of fiber alignment in PSR images was performed by coherency analysis, which indicates the overall percentage of parallel aligned collagen fibers (Fig. 1*F*). Only in collagens of IHD (*P* = 0.0402) statistically significant increases in coherency were found while collagens were more randomly oriented in HCM (*P* = 0.6226) and DCM (*P* = 0.5273) as controls.

### Immunofluorescence Staining Display the Complexity of Heart Tissues

Masson’s trichrome and PSR staining are not specific for collagen subtypes. Therefore, IF staining was performed to particularly identify and localize COL I. IF images of control myocardium, IHD, HCM, and DCM identified COL I (yellow), αSMA (red), and nuclei (blue) in all tissues (Fig. 1*D*). Different COL I morphologies were observed depending on the origin of the tissue and disease. Little amounts of COL I were detected in the control myocardium, whereas an increased number of parallelly aligned COL I fibers was observed in IHD, HCM, and DCM. Colocalization of αSMA with cell nuclei was shown in IF images visualized by purple colors. Area quantification of COL I in IF images (Fig. 1*G*) showed statistically significant increased amounts of collagen fibers in IHD (*P* = 0.0239), HCM (*P* = 0.0439), and DCM (*P* = 0.0308). For αSMA identified by IF imaging (Fig. 1*H*), a statistically significant increase in DCM was found (*P* = 0.0269) while increases in IHD (*P* = 0.1899) and HCM (*P* = 0.2583) were not statistically significant.

### Raman Imaging Allows Marker-Indepentent Visualization of Tissue Structures

**Figure 2.**
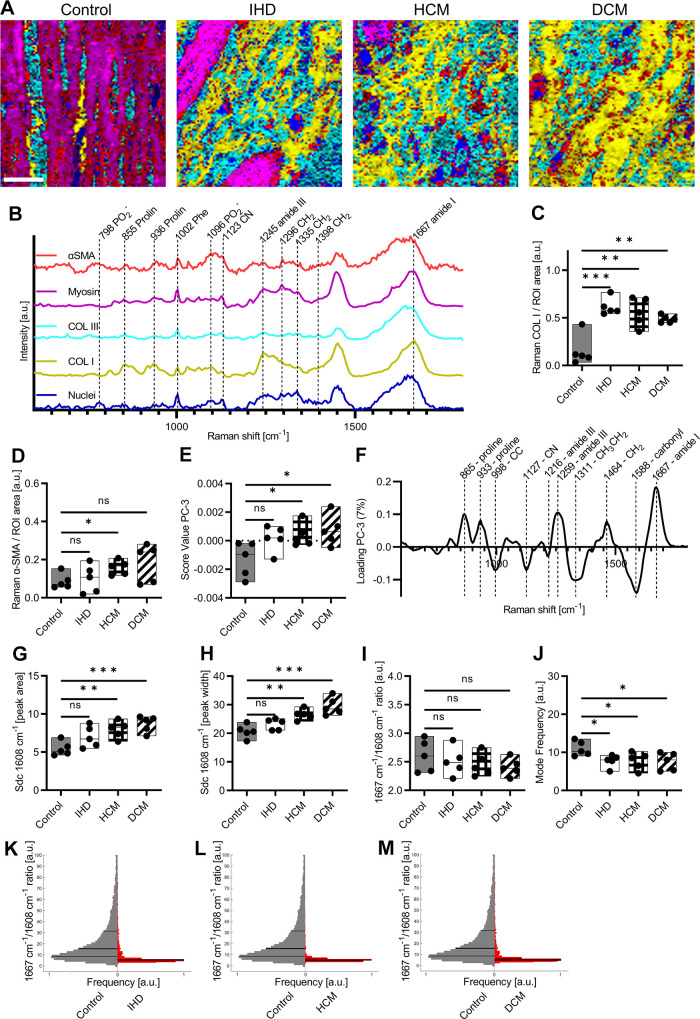
Raman imaging and spectral deconvolution (Sdc) allows marker-independent analysis of myocardium and different cardiomyopathies. *A*: True Component Analysis (TCA) images of control, IHD, HCM, and DCM. Scale bar = 20 µm. *B*: spectra identified by TCA: nuclei (blue), myosin (pink), COL III (turquoise), COL I (yellow), and αSMA (red). *C*: quantification of the amount of COL I based on Raman images normalized by the whole tissue area. *D*: quantification of the amount of αSMA based on Raman images normalized by the whole tissue area. *E*: score value analysis of PC-3 from COL I Raman spectra from control myocardium and cardiomyopathies. *F*: corresponding loading plot. *G*: peak area at 1,608 cm^−1^ calculated based on Sdc of amide I area of averaged COL I spectra from control myocardium and different cardiomyopathies. *H*: peak width at 1,608 cm^−1^ calculated based on Sdc of amide I area of averaged COL I from control myocardium and different cardiomyopathies. *I*: peak ratio of averaged COL I spectra at 1,608 cm^−1^ normalized by amide I maximum. *J*: frequency of modes from filter image ratio at 1,608 cm^−1^ normalized by amide I maximum. *K*: histogram of filter image ratio at 1,608 cm^−1^ normalized by amide I maximum (1,667 cm^−1^) of control and IHD. Lines in the histogram represent the 25, 50, and 75 percentiles while the widest position of the histograms is displaying the mode. *L*: histogram of filter image ratio at 1,608 cm^−1^ normalized by amide I maximum (1,667 cm^−1^) of control and HCM. *M*: histogram of filter image ratio at 1,608 cm^−1^ normalized by amide I maximum (1,667 cm^−1^) of control and DCM. Statistical analysis: *t* test, *n* = 5, *P < 0.05, **P < 0.01, ***P < 0.005, ns: not significant. COL I, collagen type I; COL III, collagen type III; DCM, dilated cardiomyopathy; HCM, hypertrophic cardiomyopathy; IHD, ischemic heart disease.

Although histological and IF staining can identify fibrotic heart diseases based on collagen staining, these methods are time-consuming and require the usage of expensive chemicals. Consequently, marker-independent RMS was implemented to characterize the biomolecular composition of the myocardium and different cardiomyopathies. Raman maps of myocardium and infarcted areas were analyzed by True Component Analysis (TCA), a type of multivariate data analysis. Raman imaging and TCA (Fig. 2*A*) identified and localized five different components in myocardium tissues based on their spectral fingerprint (Fig. 2*B*). Peaks related to DNA (798, 1,096 cm^−1^) (31), could define the structures, that were detected in one component of the TCA (blue). Another component showed peak assignments reported before to proline in COL I (yellow) located at 855 and 936 cm^−1^ (32, 33). The Raman spectra of collagen type III (COL III, turquoise) shared many spectral features with COL I, however, exhibited increased intensities at 1,123 and 1,296 cm^−1^, and representatives for CN and CH_2_ (32, 34) displayed a shifted amide III peak from 1,245 to 1,248 cm^−1^ (35). In contrast, myosin (pink) was assigned by its morphology and increased Raman intensities at 827 and 853 cm^−1^ representatives for C-C in proline and ring-breathing mode in tyrosine (33, 36). Based on in-house Raman measurements validated by colocalization of IF signals, αSMA was assigned to the fifth spectral component identified by TCA (red) (22). The potential of RMS being utilized for noninvasive, and marker-independent imaging of tissue structures was identified as Raman images and IF images showed similar morphological features and comparable results in the quantification of the amounts of COL I and αSMA. Quantification of COL I by Raman imaging (Fig. 2*C*) identified statistically significant increases in all myopathies [IHD (*P* = 0.0005), HCM (*P* = 0.0032), and DCM (*P* = 0.0019)]. For αSMA detected by Raman imaging (Fig. 2*D*), a statistically significant increase in the amount was found in HCM (*P* = 0.0186), whereas the tendency of increase was found in DCM (*P* = 0.0758). Similar amounts of αSMA compared with controls were found in IHD (*P* = 0.7363).

### PCA Identifies Differences Between Healthy Myocardium and Cardiomyopathies

To determine the potential of RMS for assessment of different pathological alterations in myocardium, Raman spectra of COL I were extracted from Raman images and subjected to PCA to further investigate similarities or differences between the diseases. PC-3 score values (Fig. 2*E*) revealed statistically significant increases in HCM (*P* = 0.0330) as well as DCM (*P* = 0.0232) compared with control myocardium, whereas for IHD (*P* = 0.0642) only a tendency toward increased score values was found. The shifts in the score values were explained by the corresponding loading plot (Fig. 2*F*), which mainly indicated changes in the amide I region (1,550–1,720 cm^−1^) responsible for alteration in the secondary structure of proteins. In COL I, the secondary structure is defined mainly by α-like helices, β-sheets, β-turns, and random coils (disordered) (37, 38). Further COL I structure-related peaks were found in the loadings located at 1,216 and 1,259 cm^−1^, indicating alterations in the amide III region (39, 40). Shifts located at 1,127, 1,311, and 1,464 cm^−1^ were representatives for C-N vibrations (41), CH_3_CH_2_ twisting modes of collagen (42), and CH_2_ deformation (40). Peaks at 865 and 933 cm^−1^ could be assigned to proline (32, 33).

Furthermore, a PCA excluding the control spectra was performed to identify molecular alterations across the diseases. However, score value analysis was not able to identify statistically significant differences in Col I composition (Supplemental Fig. S1*A*; https://doi.org/10.6084/m9.figshare.23292443).

### Fibrotic COL I Alterations in Cardiomyopathies Are Identified by the Raman Marker at 1,608 cm*^−^*^1^

Spectral deconvolution of the amide I region enabled in detailed analysis of the secondary structure of COL I in control myocardium and different cardiomyopathies. COL I average Raman spectra were cropped to the amide I region (1,508–1,780 cm^−1^) and normalized to 1 for better comparability. By spectral deconvolution, five substructural peaks located at 1,562, 1,588, 1,608, 1,637, and 1,667 cm^−1^ could be identified based on the shape of the amide I region and literature (22, 27). The subpeaks were assigned to tryptophane, phenylalanine, tyrosine, β-sheets as well β-turns (37, 38). In Supplemental Fig. S1, *B*–*E*, the amide I region of averaged COL I spectra and deconvoluted subpeaks in control myocardium, IHD, HCM as well as DCM are displayed. For all deconvolutions, the adjusted *R*^2^ values were above 0.995 indicating a sufficient fitting accuracy. Spectral deconvolution provided information about peak width and the area of substructural peaks. In HCM (*P* = 0.0047) and DCM (*P* = 0.0008), calculated peak areas at 1,608 cm^−1^ were significantly increased compared with control myocardium, whereas for IHD a tendency toward an increased area was detected (*P* = 0.0721) (Fig. 2*G*). Analysis of the peak width at 1,608 cm^−1^ showed similar results. Significant increases were found in HCM (*P* = 0.0025) and DCM (*P* = 0.0009) compared with control myocardium, whereas trends of increased widths were observed in IHD (*P* = 0.1064) (Fig. 2*H*). In addition, the peak intensity ratio of 1,608 cm^−1^ to the maximum peak of the amide I peak at 1,667 cm^−1^ was calculated. However, no significant differences were observed when comparing the peak ratio of averaged COL I spectra from control myocardium with IHD (*P* = 0.5882), HCM (*P* = 0.3527), and DCM (*P* = 0.2074) (Fig. 2*I*). Further to the spectral deconvolution of averaged spectra of COL I, whole COL I Raman maps were analyzed. Based on divided sum filter images created at 1,608 ± 10 cm^−1^ and 1,667 ± 25 cm^−1^, the peak ratio per pixel in each image was assessed. Figure 2, *K*–*M* displays the calculated histograms of peak ratios from control COL I compared with IHD, HCM, and DCM. Lines in the histogram represent the 25, 50, and 75 percentiles while the widest position of the histograms is displaying the mode. Analysis of the mode (Fig. 2*J*) showed statistically significant decreases in IHD (*P* = 0.0207), HCM (*P* = 0.0411), and DCM (*P* = 0.0349).

### RMS Identify Epigentic 5mC Alteration in Cardiomyopathies

**Figure 3.**
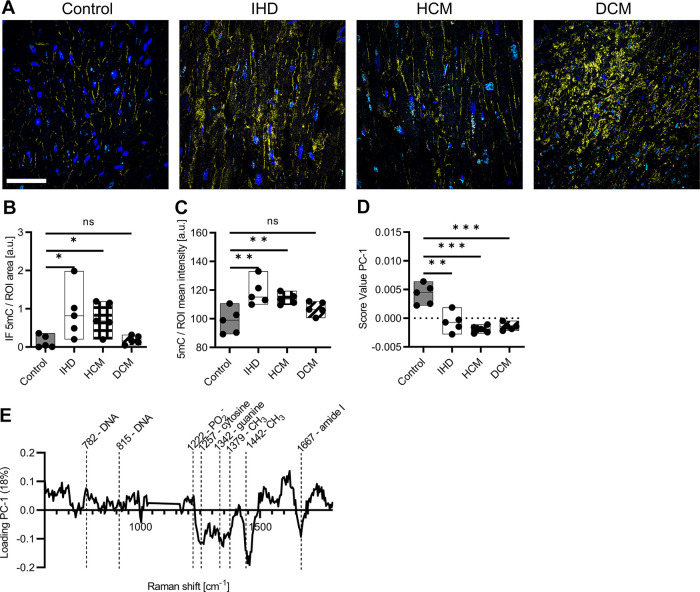
Raman imaging identifies epigenetic alterations in cardiomyopathies. *A*: IF images of 5mC staining. Colors in IF staining: Nuclei (blue), COL I (yellow), and 5mC (turquoise). Scale bar = 100 µm. *B*: quantification of the amount of 5mC based on IF images normalized by nuclei. *C*: quantification of the fluorescence intensity of 5mC. *D*: score value analysis of PC-1 from nuclei Raman spectra from control myocardium and cardiomyopathies. *E*: corresponding loading plot. Statistical analysis: *t* test, *n* = 5, *P < 0.05, **P < 0.01, ***P < 0.005, ns: not significant. COL I, collagen type I; IF, immunofluorescence.

In recent years, it has been recognized that epigenetic changes can lead to pathological tissue changes in the heart (43, 44). Therefore, 5mC IF staining was performed on nuclei of control myocardium, IHD, HCM, and DCM. IF staining identified 5mC (turquoise), nuclei (blue), and COL I (yellow), where the COL I stain served to identify the fibrotic ROIs (Fig. 3*A*). The quantification of 5mC normalized by the nuclei identified significant increases in IHD (*P* = 0.0402) and HCM (*P* = 0.0122), whereas in DCM (*P* = 0.6077) no difference was observed (Fig. 3*B*). Similar results were identified for the quantification of 5mC fluorescence intensity, where significant differences were displayed in IHD (*P* = 0.0078) and HCM (*P* = 0.0071), but not for DCM (*P* = 0.0835) (Fig. 3*C*). To further evaluate the potential of RMS in identifying diseased tissue structures, PCA was performed on extracted single spectra of nuclei. A score value analysis of PC-1 identified statistically significant differences in all IHD (*P* = 0.0023), HCM (*P* < 0.0001), and DCM (*P* = 0.0002) compared with controls (Fig. 3*D*). The corresponding loading plot (Fig. 3*E*) identified increased spectral signatures in DNA backbone located at 782, 815, and 1,222 cm^−1^ in controls (32, 33, 45). In contrast, cardiomyopathies exhibited increased spectral features located at 1,257, 1,342 cm^−1^ and representatives for cytosine and guanine (31, 46). Alternatively, the peak at 1,257 cm^−1^ could be attributed to methylations as the peaks located at 1,379 and 1,442 cm^−1^ (39, 40, 47). Furthermore, an increased signal in cardiomyopathies was shown in the amide I region at 1,667 cm^−1^, which in the case of the nuclei could indicate a change in the histone structure. As for COL I Raman spectra, a PCA of nuclei Raman spectra was calculated only for the cardiomyopathies to identify disease-related alterations, however, no statistically significant differences were observed (Supplemental Fig. S1*F*; https://doi.org/10.6084/m9.figshare.23292443).

## DISCUSSION

Myocardial fibrosis is a hallmark of pathological cardiac remodeling leading to heart failure and death. In this study, we demonstrated that noninvasive and marker-independent RMS has the potential to identify fibrotic COL I alterations and epigenetic alterations in different cardiomyopathies such as IHD, HCM, and DCM compared with control myocardium.

Histological analysis of cardiac tissue is currently the most accepted and reliable method for the investigation of pathological fibrosis ex vivo. Collagen staining with Masson’s trichrome, Movat’s pentachrome, or PSR are established methods for detecting cardiovascular fibrosis (48, 49). These stains provide a distinct contrast to differentiate collagens from their surrounding cells and connective tissues. Although these methods are very useful for the study of collagen density and structure, the staining procedure is tedious and requires large amounts of reagents (50). In addition, histopathological examination by trained pathologists can easily introduce bias and error by selecting nonrepresentative sites from the tissue biopsy, leading to poor reliability of both qualitative and quantitative outcomes. The clinical diagnosis of fibrotic diseases may also occasionally vary based on individual experience and knowledge, leading to divergent diagnostic results (51).

PSR staining is utilized to evaluate information about the condition of collagen fibers by their birefringent characteristics. It is widely reported that thicker more mature collagen fibers polarize light toward a stronger red birefringence while thinner immature fibers to a weaker green birefringence (52). The strong positive birefringence of collagen is due to the superposition of the clockwise collagen superhelix with the counterclockwise helix comprising the three polypeptide chains. In contrast, greenish-yellow collagen fibers were found possibly representing procollagen and intermediate collagen fibers (53). However, greenish collagen fibers may also indicate the presence of procollagen, intermediate, or pathological collagen fibers (54). Differential results were obtained from the quantification of mature and immature fibers in the various cardiomyopathies, indicating the involvement of distinct molecular mechanisms in their pathogenesis. In literature, shifts in birefringence were also linked to the amounts of COL I and COL III (55). The shifts compared with controls could therefore indicate different levels of collagen gene expression. Indeed, varying levels of TGF-β linked to the expression of collagens were found in cardiomyopathies before (56, 57).

In cardiac fibrosis, the differentiation of cardiac fibroblasts into myofibroblasts plays another key role. Upon exposure of the heart to inflammation, hypoxia, ischemia, or other stimulating factors, cardiac fibroblasts proliferate and differentiate into cardiac myofibroblasts expressing αSMA and secreting numerous cytokines and ECM proteins (58–61). Accordingly, αSMA was selected as the second fluorophore in IF stains. The quantification of αSMA revealed increases in the amount in all cardiomyopathies. The result of the statistically increased amount of αSMA in DCM is in line with the results of recent work (62). Although fibroblasts expressing αSMA were observed in large scars after myocardial infarction (63, 64), the absence of αSMA in interstitial fibrosis was found in some cases of pulmonary fibrosis (65, 66) but also in heart muscle (67), which might explain the rather low level of αSMA in some donors.

To overcome the obstacles of histological and IF staining including their high cost and lengthy procedures, RMS was utilized to differentiate between fibrotic and control COL I fibers. TCA-based image generation provided noninvasive and marker-independent determination and localization of major sub- and extracellular structures within the cardiac tissues and respective cardiomyopathies. Heat maps of the intensity distribution of the individual TCA components allowed further exploration of the underlying spectral information. Spectral signatures provided access to changes in molecular composition of COL I and identified fibrosis-specific peak patterns in PCA loadings. Multivariate data analysis tools allowed us to discriminate between control COL I and all different cardiomyopathies mainly by spectral alteration at positions relevant to structural information. In the loadings, peaks representative of amide I and amide III were identified. In addition, peaks attributable to differences in C-C, C-N, CH_2_, and CH_2_CH_3_ vibrations were identified, which together indicate an altered pattern in the amino acid sequence of COL I.

In a recent study, we established a Raman biomarker that allowed us to discriminate fibrotic COL I from controls in various human tissues based on spectral deconvolution of the amide I region at the position at 1,608 cm^−1^ (22). The amide I peak of COL I contains information about the amino acid composition and provides additional information about the secondary structure (27, 68). Performing spectral deconvolution on averaged COL I spectra from control cardiac and different cardiomyopathies revealed statistically significant increased peak areas as well as peak widths at 1,608 cm^−1^ in all fibrotic cases including IHD, HCM, and DCM. In addition, statistically significant differences were found when comparing the modes of peak ratio images where each image contains multiple single spectra of COL I. In fibrotic COL I, the higher peak area and width at the Raman shift of 1,608 cm^−1^ could be attributed to the amino acid tyrosine and its precursor phenylalanine (41, 69). The molecular composition of collagens consists mainly of the repeating amino acid triplet’s glycine-Xaa-Yaa, where Xaa and Yaa can constitute any amino acids (70). Most frequently, the amino acids proline and hydroxyproline occupy the positions Xaa and Yaa, but can be replaced by leucine, arginine, phenylalanine, or tyrosine (71, 72). The increase in the Raman signal at 1,608 cm^−1^ accordingly represents an alteration in the amino acid sequence of fibrotic COL I, which has been linked to shifted dissociation constants of heat shock protein 47 (HSP47) (72, 73). The collagen-specific chaperon HSP47 residing in the endoplasmic reticulum is relevant for collagen synthesis in vertebrates and is a promising therapeutic target in fibrosis, which might play an important role in the development of fibrosis (74, 75). It has been demonstrated that hampering HSP47 has the potential to improve the phenotype of different types of fibrosis such as peritoneal fibrosis as well as liver fibrosis (76, 77). Furthermore, the influence of HSP47 in myocardial infarction has been found (78, 79). Our results suggest that RMS may be useful for screening the effects of antifibrotic drugs.

In recent years it was recognized that epigenetic modifications may play another important role in the development of pathological tissue modifications such as fibrosis. The methylation of DNA and modification of the histone structure are regulators for cell proliferation and their behavior (80, 81). The most frequent epigenetic alteration is 5mC, comprising 4% of all cytosines in the human genome, which can be propagated by DNA replication mediated by DNMT1 DNA methyltransferase (82, 83). IF imaging revealed statistically significant increases of 5mC in nuclei located in fibrotic areas. Furthermore, the spectral signatures identified in a PCA of the cell nuclei can be assigned to changes in the DNA and methylation patterns implicating the potential of RMS being utilized to identify epigenetic modifications. When the increased epigenetic signals of the cardiomyopathies were considered together with the results of COL I and αSMA analyses, a consistent correlation emerged. The epigenetic modification of the DNA may cause the myofibroblasts, which normally disappear via apoptosis during normal wound healing, to develop resistance mechanisms such that they continue to secrete COL I even after the healing process has ended (14). The excessive production of COL I could then ultimately lead to pathological tissue modification.

Moreover, the findings from Raman imaging and histological analysis are intriguing, as they demonstrate the potential for distinguishing between various types of cardiomyopathies. It was observed that IHD had a higher collagen content compared with the other types of investigated cardiomyopathies. However, there was no clear correlation between the amount of collagen and molecular markers for fibrosis, suggesting that a higher collagen content does not necessarily correlate with a stronger fibrotic phenotype. This observation is particularly interesting as it may indicate a more complex relationship between collagen deposition and the development of fibrosis, which warrants further investigation in future studies.

The advancement of RMS as a benchtop method for companion diagnostics in pathology or an RMS-based endoscopic device that can be utilized during clinical surgery offers promising alternatives to conventional techniques. Such RMS-based tools enable rapid and precise real-time analysis of tissue samples without the need for prior staining or fixation. RMS provides molecular signatures of cells and tissues, identifies biomarkers and gene expression profiles, and facilitates personalized treatment approaches. In pathology, it allows for faster and more accurate diagnoses while during surgical procedures, it provides real-time diagnostics of tissue structures and margins. Despite certain technical challenges, RMS holds great potential for improved diagnostic procedures and personalized treatments.

The regulatory hurdles associated with the application of RMS as a benchtop method or endoscopic system involve fulfilling regulatory requirements and integrating the technology into clinical practice. These requirements include demonstrating the safety, effectiveness, and reliability of RMS as a diagnostic technique through extensive clinical studies and data that compare its performance to established diagnostic methods and gold standards. In addition, compliance with regulatory standards for the manufacturing, validation, and quality assurance of devices and reagents is necessary. Obtaining regulatory approvals and certification from relevant authorities to officially recognize RMS as a diagnostic procedure can be time-consuming and costly due to varying regulations across different countries or regions (e.g., EMA vs. FDA). Moreover, the integration of RMS into clinical practice requires training medical personnel and pathologists to properly apply the technique and interpret the data, along with the development and implementation of standardized protocols and guidelines for consistent and reliable application. Close collaboration among researchers, manufacturers, regulatory authorities, and medical professionals is necessary to overcome these challenges and fully exploit the potential of this innovative diagnostic technology.

### Conclusions

This study highlighted the potential of RMS as a diagnostic tool to evaluate and identify fibrotic COL I and epigenetic DNA modifications in different human cardiomyopathies. RMS allowed discriminating pathological COL I in fibrotic IHD, HCM, and DCM by spectral deconvolution of the amide I band. Moreover, PCA allowed identifying epigenetic 5mC modifications in the DNA of diseased patients. This molecularly sensitive approach enables the monitoring of pathological tissue changes and, at the same time, gives insights into the pathogenesis of the disease. Whereas only a small selection of cardiac fibrotic diseases has been studied, this technique may, in the future, provide pathologists with a nondestructive, marker-independent, and potentially automated method to perform fibrosis and epigenetic studies without the need for conventional time-consuming staining procedures.

## DATA AVAILABILITY

The data that support the findings of this study are available from the corresponding author upon reasonable request. 

## SUPPLEMENTAL DATA

10.6084/m9.figshare.23292443 Supplemental Fig S1: https://doi.org/10.6084/m9.figshare.23292443.

## GRANTS

This work was conducted in the framework of the Graduate School 2543/1 “Intraoperative Multi-Sensory Tissue-Differentiation in Oncology” funded by the German Research Foundation (DFG - Deutsche Forschungsgemeinschaft). Further funding was received by the Deutsche Forschungsgemeinschaft (INST 2388/64-1, INST 2388/33-1 and Germany’s Excellence Strategy, EXC 2180-390900677), the Ministry of Science, Research, and the Arts of Baden-Wuerttemberg (33-729.55-3/214 and SI-BW 01222-91), and the State Ministry of Baden-Wuerttemberg for Economic Affairs, Labour and Tourism (3-4332.62-NMI/65).

## DISCLOSURES

No conflicts of interest, financial or otherwise, are declared by the authors.

## AUTHOR CONTRIBUTIONS

A.N. and K.S.-L. conceived and designed research; L.B. performed experiments; L.B. and J.M. analyzed data; L.B., I.A.M.-M., F.F., J.M., and K.S.-L. interpreted results of experiments; L.B. prepared figures; L.B. drafted manuscript; I.A.M.-M., S.L.L., A.N., F.F., J.M., and K.S.-L. edited and revised manuscript; L.B., I.A.M.-M., S.L.L., A.N., F.F., J.M., and K.S.-L. approved final version of manuscript.
